# What the Eye Sees, the Mind Rejects: Implicit Visual Processing of Food Images in Anorexia Nervosa

**DOI:** 10.1002/erv.3210

**Published:** 2025-05-19

**Authors:** M. Dimakopoulou, T. Ciorli, M. Pyasik, C. Andriulli, F. Bevione, M. Martini, G. Abbate Daga, L. Pia

**Affiliations:** ^1^ SAMBA (SpAtial, Motor & Bodily Awareness) Research Group Department of Psychology University of Turin Turin Italy; ^2^ Laboratory of Cognitive Neuroscience Department of Languages and Literatures, Communication, Education and Society University of Udine Udine Italy; ^3^ AOU Città della Salute e della Scienza di Torino Department of Neuroscience University of Turin Turin Italy; ^4^ Neuroscience Institute of Turin (NIT) Turin Italy

**Keywords:** Anorexia Nervosa, food processing, implicit attitudes, visual awareness

## Abstract

**Objective:**

This study aims to explore the role of implicit visual processing in reinforcing maladaptive eating behaviours in Anorexia Nervosa‐restricting subtype (AN‐R), focussing on how high‐ and low‐calorie food stimuli are processed at different stages.

**Method:**

Thirty‐two AN‐R females and 36 healthy controls participated. Using a combination of novel paradigms in the field, the study employed: Breaking Continuous Flash Suppression (bCFS) for unconscious detection, Binocular Rivalry (BR) for perceptual dominance, and the Food Preference Approach‐Avoidance Task (FP‐AAT) for subconscious food associations.

**Results:**

AN‐R individuals exhibited prolonged perceptual dominance for high‐calorie foods but simultaneously displayed stronger implicit avoidance tendencies towards these foods. Notably, the perceptual advantage correlated with heightened interoceptive awareness, while avoidance was linked to body dissatisfaction and difficulty tolerating bodily sensations. Conversely, healthy females showed the opposite pattern, implicitly approaching high‐calorie foods while avoiding low‐calorie foods, suggesting a more adaptive integration of food‐related cues.

**Conclusions:**

This study provides novel insights into the complex role of high‐calorie foods in AN, highlighting whether and how different aspects of implicit visual processing influence eating behaviours, and underscoring the need for targeted interventions incorporating implicit cognitive mechanisms to address visual processing biases and support AN recovery.

**Summary**

- First study to closely examine visual processing in AN across three distinct stages (unconscious detection, perceptual dominance, and subconscious associations) using novel paradigms.
- AN‐R individuals show paradoxical responses to high‐calorie foods, with prolonged visual dominance but simultaneous implicit avoidance, reinforcing restrictive eating patterns.
- Findings suggest that high‐calorie foods act as arousal triggers in AN, linking visual biases to interoceptive awareness, body dissatisfaction, and maladaptive eating behaviours, highlighting new intervention targets for AN recovery.

## 1 Introduction

Anorexia nervosa (AN) is a severe mental health condition that predominantly affects adolescent females, with high morbidity and mortality (Solmi et al. 2024). The nature of such a disorder is complex and multifactorial because of several biological, social, and cognitive variables that are concurrently involved in its genesis and maintenance (APA 2025). Its core features include an intense drive for thinness associated with highly dysfunctional beliefs about body image and food. Regarding food, individuals with AN engage more cognitively and emotionally with food‐related stimuli than healthy individuals (Joos et al. 2011; Uher et al. 2004). Subjects with AN typically avoid high‐energy‐dense food (e.g., fats, carbohydrates, or sweets) while attributing excessive importance to low‐energy‐dense food (Hetherington and Rolls 1991; Jauregui Lobera and Bolanos Rios 2009). Such maladaptive eating attitudes are maintained through different compensatory mechanisms like excessive reduction of food intake (restricting subtype of AN) or binge‐purge behaviours (binge eating‐purging subtype of AN). Neurobiological models of AN suggest that these behaviours derive from aberrant responses to food involving craving, reward, and attentional processing (Bronleigh et al. 2022). Research using pictorial food stimuli has explored how AN individuals process food categories with different caloric content, given vision's key role in detecting nutritional sources. However, findings remain inconclusive, complicating the clinical approaches (Giel et al. 2011). Indeed, some studies indicate a strong bias towards high‐calorie foods, interpreted as a response to threat or appetitive value (Neimeijer et al. 2017; Shafran et al. 2007, 2008; Smeets et al. 2008), while others report disengagement from food‐related cues (Jonker et al. 2019) or a preference for low‐calorie foods (Veenstra and de Jong 2012).

Building on these considerations, for the first time, we closely examined different aspects of the visual processing of highly palatable and low‐energy‐density foods in AN, focusing exclusively on the restricting subtype. While previous studies have either focused on AN‐R (Neimeijer et al. 2017; Veenstra and de Jong 2012) or included both subtypes (Jonker et al. 2019; Shafran et al. 2007), their behavioural differences, despite a shared focus on extreme dieting, may explain conflicting findings. To account for these differences, we recruited only AN‐R subjects, as their stable responses to food exposure make them more suitable for studying visual food processing (Gilon Mann et al. 2018). Moreover, since food intake is also influenced by lower‐level cognitive processes (Bargh 1994), understanding these mechanisms can be fundamental to identify (potentially altered) functions operating beyond direct conscious control. Therefore, we focused on more implicit aspects of visual processing: visual awareness and automatic associations. Regarding visual awareness, we addressed the breaking‐Continuous Flash Suppression (bCFS; Jiang et al. 2007) and the Binocular Rivalry (BR; Tong et al. 2006) paradigms. The bCFS measures the time an invisible stimulus takes to overcome interocular suppression, with faster detections indicating differential unconscious processing (Ciorli et al. 2025; Stein 2019). Instead, the BR assesses perceptual dominance by simultaneously presenting competing stimuli to each eye, where longer perceived durations reflect prioritisation within conscious visual awareness. Concerning implicit associations, we capitalised on a variation of the Implicit Association Test (IAT; Greenwald et al. 2003). Specifically, we administered the Food Preferences Approach‐Avoidance Test (FP‐AAT; Rinck and Becker 2007) to assess the automatic approach‐avoidance tendencies towards food categories based on perceived calorie content. Finally, we explored correlations between these experimental paradigms and clinical questionnaires related to AN psychopathology. Compared to healthy participants, we hypothesised that food categories with different caloric content would differentially affect visual awareness in subjects with AN‐R, with high‐calorie food images gaining greater relevance, leading to faster access and dominance in awareness. We also predicted automatic avoidance tendencies for high‐calorie foods and expected these patterns to correlate with clinical measures, particularly aspects of body image.

## 2 Materials and Methods

### 2.1 Participants

Thirty‐two individuals affected by AN‐R were recruited from the Eating Disorder Center of “Città della Salute e della Scienza” Hospital at the University of Turin, from January 2021 to December 2024. The sample size was estimated through an a priori power analysis for a repeated‐measures ANOVA with a between‐subjects factor, based on the effect size *η*_*p*_^2^ = 0.18 reported by Ciorli and colleagues (Ciorli et al. 2024). With two groups, alpha = 0.05% and 90% of statistical power, the required sample size was determined to be *N* = 32. The inclusion criteria were as follows: (a) female; (b) diagnosis of AN‐R assessed by an experienced psychiatrist through the Structured Clinical Interview for Diagnostic and Statistical Manual of Mental Disorders 5th edition (DSM‐5); (c) written consent for ethical approval of the study (conducted according to the principles of the Declaration of Helsinki and approved by the ethical committee of the A.O.U. City of Health and Science Prot. N° 0006434). Clinical Assessment was generally conducted at admission. The control group (HC) comprised 36 healthy females recruited from the University of Turin's database. These individuals had a regular BMI, no history of Eating Disorders (ED), and provided written informed consent for participation (study approved by the University of Turin ethical committee, Prot. N° 100960). All participants were Italian speakers, aged 18–30, with normal or corrected‐to‐normal vision, and no history of substance use or mental disorders. Demographic comparisons between the AN‐R and HC groups are presented in Table 1.

**TABLE 1:** Comparison of demographic characteristics and psychological measures between individuals with AN‐restricting subtype (AN‐R) and healthy controls. Values are presented as means *(M)* and Standard Deviations (SD). Group comparisons were performed using ANOVA, with *t*‐values, degrees of freedom (df), and *p*‐values reported. Statistically significant differences (*p* < 0.05) are highlighted.

	Subjects with AN‐R		Healthy controls (*n* = 36)		Group comparisons		
Variables	*M*	SD	*M*	SD	*t*	df	*p*
Age (years)	23.16	2.23	24.89	2.93	2.60	1	0.011
BMI (kg/m^2^)	15.10	1.67	20.85	1.67	14.07	1	< 0.001
Educational level (years)	13.57	1.79	16	2.13	4.85	1	< 0.001
EDI‐2							
Drive for thinness	14.07	6.34	3.14	5.08	−7.66	1	< 0.001
Bulimia	0.78	1.52	1.47	2.39	1.32	1	0.20
Body dissatisfaction	14.75	5.64	6.33	5.32	−6.12	1	< 0.001
Ineffectiveness	14.18	5.56	4.03	4.37	−8.18	1	< 0.001
Perfectionism	5.00	4.11	4.30	3.12	−0.77	1	0.45
Interpersonal distrust	7.82	4.36	3.06	3.82	−4.65	1	< 0.001
Interoceptive awareness	12.79	6.89	4.36	5.92	−5.25	1	< 0.001
Maturity fears	9.25	5.54	4.44	4.05	−4.01	1	< 0.001
Asceticism	7.79	4.78	2.50	1.80	−6.16	1	< 0.001
Impulse regulation	6.14	4.84	2.30	4.08	−3.44	1	< 0.001
Social insecurity	10.39	3.38	4.06	3.77	−6.63	1	< 0.001
EDE‐Q							
Restraint	3.19	1.70	1.09	1.07	−6.05	1	< 0.001
Eating concern	3.14	1.25	0.89	1.10	−7.72	1	< 0.001
Shape concern	4.54	1.05	1.84	1.58	−7.88	1	< 0.001
Weight concern	3.92	1.28	1.39	1.35	−7.69	1	< 0.001
Global score	3.70	1.09	1.30	1.12	−8.64	1	< 0.001
MAIA‐2							
Noticing	2.30	0.91	2.96	0.94	−0.10	1	0.92
Not distracting	2.52	0.83	2.54	0.65	0.14	1	0.90
Not worrying	2.00	0.86	2.56	0.98	2.28	1	< 0.05
Attention regulation	1.60	0.71	2.43	0.94	3.67	1	< 0.05
Emotional awareness	3.17	2.43	3.03	1.06	−0.30	1	0.76
Self‐regulation	1.60	1.01	2.32	1.16	2.42	1	< 0.05
Body listening	1.50	0.93	2.34	1.38	2.60	1	< 0.05
Trusting	1.00	0.92	3.02	1.34	6.39	1	< 0.05
BSQ‐34	118.27	48.62	78.94	33.08	−3.76	1	< 0.001
STAI							
State anxiety	57.89	11.91	41.19	11.15	−5.77	1	< 0.001
Trait anxiety	59.86	9.73	47.22	11.00	−4.80	1	< 0.001
BDI‐II							
Cognitive	16.61	6.53	6.19	5.18	−6.80	1	< 0.001
Somatic	13.65	5.47	5.64	4.49	−6.14	1	< 0.001
Total score	30.26	11.59	11.83	9.40	−6.70	1	< 0.001

### 2.2 Clinical Assessment

We administered the following questionnaires: Eating Disorder Inventory (EDI‐2), Eating Disorder Examination Questionnaire (EDE‐Q), Multidimensional Assessment of Interoceptive Awareness‐2 (MAIA‐2), Body Shape Questionnaire‐34 (BSQ‐34), State‐Trait Anxiety Inventory (STAI), and Beck Depression Inventory‐II (BDI‐II). See Table 1 for a comparison of psychological measures between AN‐R individuals and HC. Due to space limitations, a detailed description is reported in the supplement (Supporting Information S1: Section 1).

### 2.3 Experimental Tasks

#### 2.3.1 Stimuli and Set Up

Stimuli were obtained from the Food‐pics Database (Blechert et al. 2019). We selected six high‐calorie (pistachio nuts, doughnuts, chocolate biscuits, hamburger, pizza, lasagna) and six low‐calorie (zucchini, tomato, mushrooms, green beans, mixed steamed vegetables, apple) items, which were standardized to a circular frame. Stimuli were then superimposed onto an image of a plate contour to ensure symmetrical binocular fusion. Although the stimuli were already equated in the database, low‐level features (brightness, contrast, and spatial frequency) were extracted and analysed using a one‐way ANOVA to check for potential differences (*p*s > 0.05). The experiment was created using MATLAB (Release, R2021b n.d.) and Psychtoolbox. Stimuli were presented on a BenQ Monitor (1920 × 1080 pixels resolution, 120 Hz, 24″) positioned 57 cm in front of the participants. Participants' heads were placed on a homemade chinrest connected to a stereoscope, adjusted to maintain stable binocular vision.

#### 2.3.2 Breaking‐Continuous Flash Suppression (bCFS)

**FIGURE 1:**
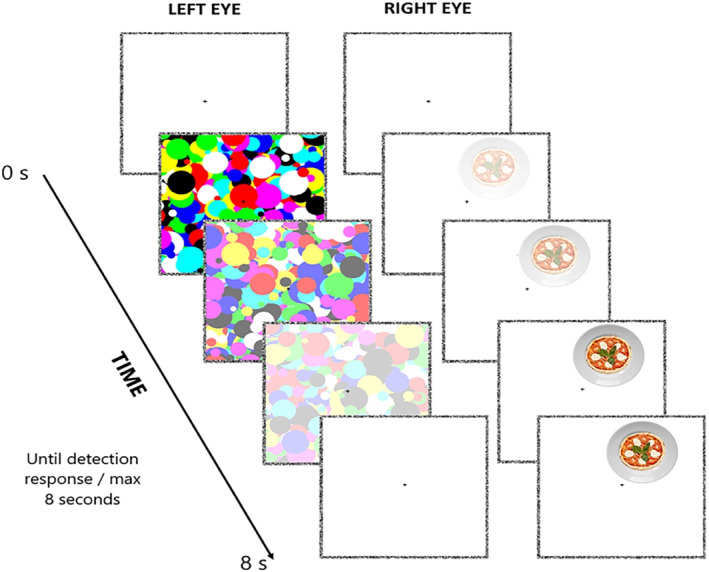
Schematic representation of a breaking Continuous Flash Suppression (bCFS) trial. After a 1‐s inter‐trial interval, a high‐contrast dynamic Mondrian mask was shown to one eye, and its transparency increased from 0% to 100% within 1–7 s after trial onset. Simultaneously, a target food stimulus (high‐ or low‐calorie) was presented to the other eye, with its transparency decreasing from 100% to 0% during the first second of the trial. Participants maintained central fixation and were instructed to detect the position of the emerging target (top or bottom of the display) as quickly and accurately as possible by pressing the corresponding arrow key. Each trial ended upon response or after a maximum of 8 s.

The bCFS measures the time an initially invisible target stimulus presented to one eye requires to break the conscious suppression induced by another masking stimulus administered to the other eye. The rationale is that stimuli overcoming suppression faster are prioritised before visual awareness (Stein 2019). In other words, bCFS relies on a direct index of conscious perception to measure differential unconscious processing, namely, how long a stimulus takes to be consciously detected. Here, the target stimuli were high‐ and low‐calorie food images covered by a dynamic (10 Hz) high‐contrast Mondrian‐pattern mask. Both the stimulus (3.3° × 3.3°) and the mask (9.5°) were presented inside a fusion square (9.5° × 9.5°, one per eye, each at 5.1° from the centre) that was created with noise pixels (width 0.2°). The background screen was black, while the area of the square was white with a black fixation cross in the centre. In each trial, the target was displayed by decreasing its transparency from 100% to 0% during the first second and presented at the top or the bottom of the square with a random horizontal jitter. Simultaneously, the transparency of the mask was linearly increased from 0% to 100% within 7s (starting after the first second of the trial). Participants were required to maintain eye fixation on the cross to prevent active target searching. They were also instructed to avoid eye‐blinking or closing one eye throughout the whole procedure. Their task was to detect the target's position (top or bottom of the fusion square) as fast and accurately as possible by pressing the corresponding arrow key (i.e., top‐position: up arrow key, bottom‐position: down arrow key), even if they only had a strong sense that something beyond the mask was present, to prevent conscious perception. Each trial ended with the localization response or after 8 s (with 1s of ITI). Following eight practice trials, the experiment began. Target eye presentation (left/right), location (top/bottom), and condition (high/low calorie) were counterbalanced across a total of 288 trials (36 trials for each condition). Short eye‐resting breaks were provided after trials 96 and 192 (see Figure 1).

#### 2.3.3 Binocular Rivalry (BR)

**FIGURE 2:**
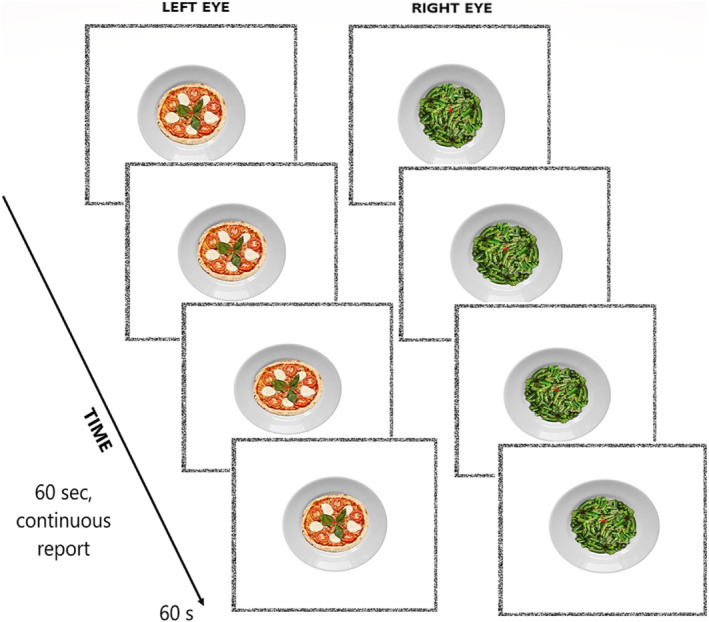
Schematic representation of a Binocular Rivalry (BR) trial. Participants viewed a high‐calorie stimulus presented to one eye and a low‐calorie stimulus to the other eye for 60 s. They were instructed to maintain central fixation and to continuously report their dominant percept by pressing the corresponding arrow key: left arrow for high‐calorie predominance, right arrow for low‐calorie predominance, and up arrow for no clear predominance. After each trial, participants had a 10‐s break, with a 60‐s eye‐resting break provided after 12 trials.

BR measures the perceptual dominance of a stimulus presented to one eye, competing for conscious representation with another stimulus presented to the other. Typically, conscious perception alternates between stimuli since the brain cannot simultaneously unify different images seen by each eye. The rationale is that stimuli reported for a longer time are prioritised within perceptual awareness. A high‐calorie stimulus was presented to one eye, whereas a low‐calorie stimulus was presented to the other (counterbalancing eye‐calorie pairs) for 60 s. Stimuli were displayed within squared frames (7.2° × 7.2°) on a black background, made of white and black pixels (width 0.2°), positioned 5° from the centre (one on the left, one on the right), with a black central fixation cross. Participants were instructed to fixate on the cross, avoiding eye‐blinking or closing one eye, and to continuously report the dominant stimulus throughout the trial. After four practice trials, the experiment began. Each trial lasted 60 s. Participants pressed the corresponding arrow key to indicate their perception each time it changed: the right arrow if they perceived more than 50% of the low‐calorie stimulus (low‐calorie predominance), the left arrow for more than 50% of the high‐calorie stimulus (high‐calorie predominance), or the up arrow if no stimulus was dominant. If no key was pressed, the trial continued automatically for the full 60 s without interruption. Each condition included 24 randomized trials (12 with each stimulus presented to the left and right eye). A ten‐second break followed each trial, with a 60‐s eye‐rest break provided after 12 trials (see Figure 2).

#### 2.3.4 Food Preferences Approach‐Avoidance Test (FP‐AAT)

We utilised the FP‐AAT to assess the implicit attitudes towards different food categories by quantifying participants' attraction or repulsion to specific stimuli. The stimuli included eight images of hypercaloric foods (e.g., pizza, french fries, chips, chocolate), eight images of hypocaloric foods (e.g., fruits, white meat), and eight neutral stimuli (numerical digits such as 7002 and 7010). Each category of stimuli was presented within a square or round frame, with the frame shape always serving as the target. Before each trial, a red “X” fixation point appeared at the centre of the screen. Participants clicked on it to initiate the trial and were then instructed to pull the mouse towards themselves or push it away, depending on the frame shape (circle or square). The underlying assumption was that the semantic properties of the task‐irrelevant stimulus (e.g., calorie content) carried a specific valence (avoidance or attraction), potentially influencing response times depending on whether the response action (pushing/pulling) aligned or conflicted with the stimulus category. For instance, participants who preferred hypercaloric foods were expected to pull the mouse faster than push it away. Participants were instructed to move the mouse cursor until it reached the upper or lower edge of the screen (i.e., to digitally “touch” the top or bottom screen border). As they performed the movement, the stimulus progressively expanded (pull) or shrank (push) to enhance the visual feedback associated with the motor action. During the practice session, if an incorrect response was given, the word “error” appeared at the centre of the screen.

**FIGURE 3:**
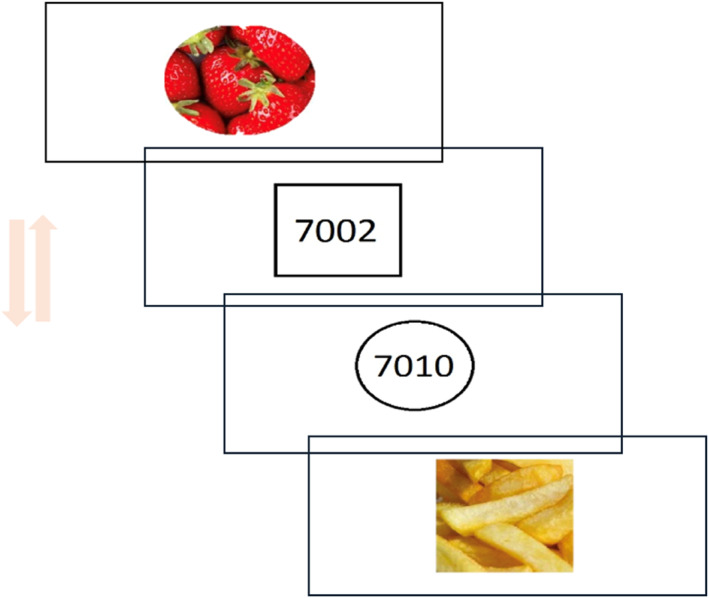
Schematic representation of a Food Preferences Approach‐Avoidance Task (FP‐AAT) trial. At the start of each trial, a red “X” appeared at the centre of the screen, which participants clicked to initiate the trial. A stimulus image (hypercaloric food, hypocaloric food, or neutral digit) then appeared within either a square or a circle frame. Participants were instructed to pull the mouse towards themselves or push it away based on the frame shape (circle or square), while ignoring the content of the image. As participants moved the mouse, the stimulus progressively expanded (pull) or shrank (push) to reinforce the movement perception. Each trial ended once the movement was completed, with reaction time recorded from trial onset.

The experiment consisted of 20 practice trials and 80 experimental trials. Each of the 24 stimuli (8 hypercaloric, 8 hypocaloric, 8 neutral) was presented twice, once in a square frame and once in a circle frame, for a total of 48 trials. Additional stimulus repetitions were included to reach 80 trials, with an approximately equal distribution across hypercaloric, hypocaloric, and neutral categories (see Figure 3).

## 3 Statistical Analysis

### 3.1 Clinical Assessment

A series of *T*‐tests were conducted with Group (AN‐R, HC) as the independent variable and score on each questionnaire as the dependent variable.

### 3.2 Experimental Tasks

A repeated measures ANOVA or a Mann‐Whitney *U* Test (for non‐normally distributed data, Shapiro‐Wilk *p* < 0.05) was conducted with Group (AN‐R, HC) and Calorie Content (High, Low) as the independent variables, and RTs (bCFS or BR) or D‐scores (FP‐AAT) as the dependent variable. Additionally, Spearman correlations were performed between the dependent variables and the scores from the clinical assessment questionnaires.

## 4 Results

### 4.1 Clinical Assessment

*T*‐tests revealed significantly higher scores on almost all of the clinical assessment questionnaires for the AN‐R group compared to the HC group (*p* < 0.05). Due to space constraints, the detailed results are provided in the supplement (Supporting Information S1: Section 2).

### 4.2 Experimental Tasks

#### 4.2.1 Breaking‐Continuous Flash Suppression (bCFS)

None of the participants reported unstable binocular perception, and trials with response times (RTs) < 300 ms were discarded, as they indicated failed suppression (1.7% of the trials). Mean RTs for corrected responses were calculated, and values ± 2.5 SD from each group mean (3%) were replaced with the respective group mean. The Mann‐Whitney *U* Test on Group (AN‐R, HC) x Calorie Content (High, Low) revealed no group differences for both high‐calorie (*u* = 425 *z* = −0.556, *p* = 0.578; AN‐R mean = 3.625 SE = ± 0.623; HC mean 2.849 = ± 0.198) and low‐calorie (*u* = 414 *z* = 0.714 *p* = 0.474; AN‐R mean = 3.569 SE = ± 0.593; HC mean 2.774 = ± 0.196) food.

#### 4.2.2 Binocular Rivalry (BR)

**FIGURE 4:**
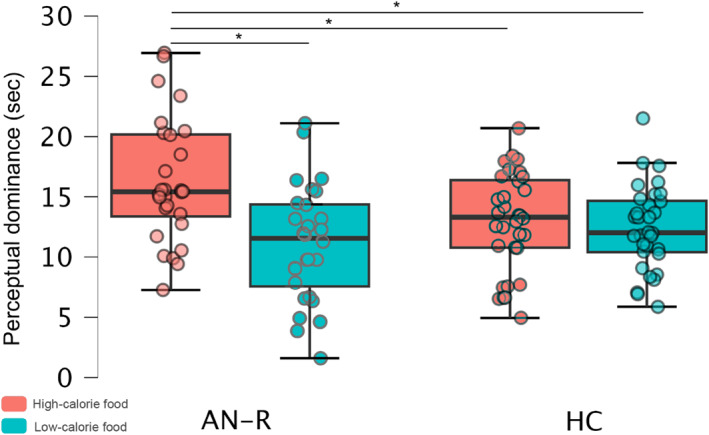
Box plots comparing dominance times (BR) of high and low‐calorie foods in the AN‐R group and healthy controls. The box represents the interquartile range (IQR, 25th–75th percentile), with the bold line indicating the median. Whiskers extend to 1.5 × IQR, and individual data points are overlaid. Significant differences between groups are marked with an asterisk (*p < 0.05). Horizontal brackets indicate statistical comparisons.

No participants reported extremely short dominance times or unstable binocular perception. Mean RTs for high‐ and low‐calorie stimuli were calculated, while mixed dominance times were excluded from the analysis. Mean RTs for corrected responses were computed, and values that deviated by more than ± 2.5 SD from each group mean (2%) were substituted with the respective group mean. A repeated measures ANOVA on Group (AN‐R, HC) x Calorie Content (High, Low) revealed a main effect of calorie content (F_(1,58)_ = 14.00, *p* < 0.001, *η*_*p*_^2^ = 0.19) with longer RTs for high‐calorie (mean = 14.60, SE = ± 0.06) compared to low‐calorie (mean = 11.75, SE = ± 0.54) food. The interaction between Group and Calorie Content was also significant (F_(1,58)_ = 9.32, *p* = 0.003, *η*_*p*_^2^ = 0.14). Post‐hoc Bonferroni's test revealed that RTs for high‐calorie food in the AN‐R group (mean = 16.27, SE = ± 0.87) were significantly longer (*p* < 0.05) than for any other condition: low‐calorie food in the AN‐R group (mean = 11.09, SE = ± 0.79), high‐calorie food in the HC group (mean = 12.94, SE = ± 0.82), and low‐calorie food in the HC group (mean = 12.41, SE = ± 0.73). See Figure 1 for a graphical representation of these results. The main effect of the group was not significant (*p* > 0.05). A sensitivity power analysis on the interaction effect size revealed that we had 80% power to detect an effect size (*η*_*p*_^2^ = 0.14) as observed (*η*_*p*_^2^ = 0.14). For the correlation analysis, RTs for high‐calorie food in the AN‐R group were positively correlated (*r* = 0.51, *p* = 0.02) with the Body Listening Scale of MAIA‐2 (see Figure 4).

#### 4.2.3 Food Preferences Approach‐Avoidance Test (FP‐AAT)

**FIGURE 5:**
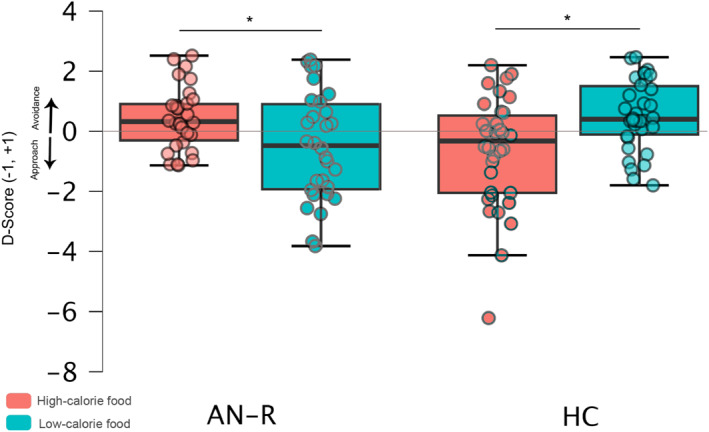
Box plots comparing FP‐AAT D‐scores of high and low‐calorie foods in the AN‐R group and healthy controls. The box represents the interquartile range (IQR, 25th–75th percentile), with the bold line indicating the median. Whiskers extend to 1.5 × IQR, and individual data points are overlaid. Significant differences between groups are marked with an asterisk (*p < 0.05). Horizontal brackets indicate statistical comparisons. Scores > 0 indicated avoidance tendencies while scores < 0 indicated approach tendencies.

A D‐score was calculated by subtracting the median push RT from the median pull RT for each stimulus group and dividing it by the pooled Standard Deviation (SD). A positive score indicated a preference for pushing (i.e., avoidance tendencies). D‐scores that deviated by ± 2.5 SD from each group's mean (1%) were replaced by the respective group's mean. A repeated measures ANOVA on Group (AN‐R, HC) x Calorie Content (High, Low) revealed a significant Group x Calorie Content interaction (F_(1,62)_ = 15.02, *p* < 0.001, *η*_*p*_^2^ = 0.19). Post‐hoc Bonferroni tests showed that in the AN‐R group, the D‐score for high‐calorie food (mean = 0.04, SE = ± 0.28) was significantly (*p* = 0.02) higher than the one for low‐calorie food (mean = −0.50, SE = ± 0.27). In contrast, in the HC group, the D‐score for high‐calorie food (mean = −0.67, SE = ± 0.26) was significantly (*p* = 0.01) lower than for low‐calorie food (mean = 0.54, SE = ± 0.26). A sensitivity power analysis revealed 90% power to detect an effect size (*η*_*p*_^2^ = 0.17) smaller than the one observed (*η*_*p*_^2^ = 0.19). Moreover, a *T*‐test for single means against zero showed that the D‐score for high‐calorie food in the AN‐R group was significantly (*p* = 0.03) higher than zero. In the HC group, the D‐score for high‐calorie food was significantly (*p* = 0.04) lower than zero, whereas the D‐score for low‐calorie food was significantly higher (*p* = 0.009) than zero. For the correlation analysis, in the AN‐R group, the D‐score for high‐calorie food was positively correlated with the Noticing (*r* = 0.49, *p* = 0.02) but negatively correlated with the Not Distracting (*r* = −0.61, *p* = 0.002) scales of the MAIA‐2. In the HC group, the D‐score for low‐calorie food was negatively correlated with the Not Worrying (*r* = −0.55, *p* = 0.001) and the Trusting (*r* = −0.37, *p* = 0.04) scales of the MAIA‐2. Additionally, in the AN‐R group, the D‐score for high‐calorie food was positively correlated with the Body Dissatisfaction scale of the EDI‐2. In the HC group, the D‐score for low‐calorie food was positively correlated with the Drive for Thinness (*r* = 0.37, *p* = 0.04) and the Maturity Fears (*r* = 0.40, *p* = 0.02) scales of the EDI‐2. In contrast, the D‐score for high‐calorie food was positively correlated with Ineffectiveness (*r* = 0.40, *p* = 0.02) and Impulse Regulation (*r* = 0.38, *p* = 0.03) of the same questionnaire (see Figure 5).

## 5 Discussion

This study investigated how subjects with AN‐R process food stimuli at various cognitive stages, from initial unconscious detection (bCFS) to conscious perceptual processing (BR) and post‐perceptual automatic associations (FP‐AAT), while examining the links between these processes and clinical assessments. Our findings suggest a vigilance‐avoidance pattern where high‐calorie foods initially dominate visual processing in AN‐R but are later avoided at an automatic post‐perceptual level. This pattern correlates positively with the Body Listening scale of MAIA‐2, indicating heightened interoceptive sensibility in these individuals. Despite perceptual dominance, high‐calorie foods were implicitly avoided, correlating positively with the Noticing scale of MAIA‐2 and the Body Dissatisfaction scale of EDI‐2 but negatively with the Not Distracting scale of MAIA‐2. This indicates that AN‐R's increased visual focus on high‐calorie foods is linked to aversive bodily and emotional responses. In contrast, healthy participants approached high‐calorie foods and avoided low‐calorie foods. Approach tendencies were positively correlated with the Ineffectiveness and Impulse Regulation scales of EDI‐2, whereas avoidance of low‐calorie foods was negatively associated with the Not Worrying and Trusting scales of MAIA‐2 and positively related to the Drive for Thinness and Maturity Fears scales of EDI‐2. These findings suggest that healthy individuals may link low‐calorie foods to dieting efforts rather than natural hunger cues.

Before interpreting the experimental results, we acknowledge the clinical characteristics of the AN‐R group. AN‐R individuals reported higher anxiety, depression, and body image disturbance than HC, as shown by STAI, BDI‐II, and BSQ‐34 scores. These findings align with the literature, which consistently reports elevated anxiety and depression in AN. For instance, Jérolon and colleagues (2022) found that anxiety and depression mediate the relationship between temperament and core AN psychopathology, while Dosal et al. (2024) highlighted the complex interplay between body perception and psychological symptoms in AN.

Concerning the experimental findings, it is plausible to hypothesise that calorie content influences visual awareness, as awareness is affected by both low‐level stimulus features and higher cognitive processes (Capitao et al. 2014; Ciorli and Pia 2023, 2024; Gayet et al. 2013; Yang and Yeh 2011). To explore the unconscious‐to‐conscious visual transition, we employed the bCFS paradigm, predicting that faster responses indicate unconscious prioritisation of specific stimuli. However, we found no differences in unconscious food processing between AN‐R and HC, nor any correlation with psychological characteristics. This suggests that preattentive, unconscious processing of food stimuli remains intact in AN‐R. Although awareness and attention may be required to distinguish their meaning, particularly in terms of perceptual dominance and implicit responses (Jusyte et al. 2015). The lack of significant effects aligns with this field's current shortage of evidence. To our knowledge, this is the first study investigating unconscious food processing in AN‐R. Since the absence of evidence is not evidence of absence (Marsh 2019), our negative findings should be interpreted cautiously. Moreover, studies on healthy participants are conflicting. Lee et al. (2022) found that high‐calorie foods were prioritised, while Ciorli and colleagues (2024) observed this effect only in a satiated state. Therefore, further research is needed to better understand the mechanisms underlying unconscious food perception in AN.

To assess perceptual dominance, we administered the BR, which measures the duration a stimulus is consciously perceived as dominant while competing with another. Our results indicate that high‐calorie food images dominated longer in AN‐R individuals, aligning with previous findings in healthy participants, where emotional salience affects visual perception (Alpers and Gerdes 2007; Alpers et al. 2005). Such a conscious prioritisation of stimuli despite their negative affective connotations (i.e., they are typically associated with threat and anxiety) may be explained by heightened arousal states triggered by food stimuli in AN. Sheth and Pham (2008) demonstrated that less pleasant images dominate under high‐arousal conditions, which could apply to the anxiety‐provoking effects of high‐calorie foods in AN (Brooks and Stein 2015; Lavender et al. 2015). Further supporting this, the perceptual dominance of high‐calorie foods correlated positively with the Body Listening scale of MAIA‐2, which assesses the tendency to actively attend to bodily sensations. This suggests that AN‐R individuals, more attuned to their bodily sensations, may exhibit hypervigilance towards high‐calorie foods due to the heightened interoceptive feedback and associated arousal. Such patterns align with evidence that AN is characterised by maladaptive interoceptive processes, where heightened sensibility to bodily sensations contributes to anxiety and avoidance behaviours (Eshkevari et al. 2014). Such interoceptive processes play a key role in attentional and perceptual biases towards emotionally salient stimuli (Herbert and Pollatos 2012). While body awareness in healthy individuals supports positive food engagement (Tylka and Kroon Van Diest 2013), in AN‐R, it may instead intensify distress and reinforce food avoidance.

While bCFS and BR reflect the earliest visual processing, we measured the approach‐avoidance tendencies to reveal subconscious responses to emotionally charged stimuli by administering the FP‐AAT (De Houwer 2006). The premise of this task is that pleasant stimuli evoke an automatic approach response, while unpleasant stimuli trigger avoidance (Bradley and Lang 2007). Our results align with prior research showing that individuals with acute AN avoid unpleasant stimuli (Spring and Bulik 2014), while alternative findings have also been reported (Paslakis et al. 2016). However, in AN‐R individuals, high‐calorie foods served as “unpleasant” stimuli due to their association with body image distress, weight gain, and loss of control, suggesting that avoidance stems from negative emotional responses to these foods (Brooks and Stein 2015). In particular, the tendency to avoid hypercaloric foods was associated with altered interoception (Noticing and Not Distracting scales ‐ MAIA‐2) and increased body dissatisfaction (EDI‐2). The Noticing scale reflects an individual's heightened awareness of bodily sensations, which can amplify distress and reinforce food avoidance due to fear of weight gain (Lavender et al. 2015). This hypervigilance may increase anxiety when confronted with high‐calorie foods, strengthening the avoidance behaviours seen in AN‐R. Meanwhile, the Not Distracting scale measures a tendency to focus on negative emotions without distraction. Our findings show that AN‐R individuals, more likely to distract themselves from unpleasant bodily sensations (lower Not Distracting scores), exhibit stronger implicit avoidance of high‐calorie foods, suggesting that disengaging from internal discomfort may heighten repulsion. Recent research supports this, showing that individuals with AN who struggle with regulating interoceptive awareness, mainly through distraction strategies, tend to have more severe food avoidance (Datta and Lock 2023; Monteleone and Cascino 2021). Additionally, the Body Dissatisfaction scale of the EDI‐2, which assesses dissatisfaction with one's body, is strongly linked to food avoidance behaviours in AN (Harrison et al. 2011). In individuals with AN‐R, avoiding high‐calorie foods may implicitly serve as an attempt to control weight and prevent worsening body dissatisfaction. Conversely, healthy participants showed an automatic approach towards high‐calorie foods, correlating with the Ineffectiveness and Impulse Regulation scales of the EDI‐2, which assess self‐efficacy and emotional control, respectively. This suggests that individuals with lower self‐efficacy and poor impulse control may be more prone to disinhibited eating under stress. Boswell and Kober (2016) linked reduced impulse control to increased calorie‐dense food consumption, while Booth et al. (2018) found that impulsivity and automatic food approach bias contribute to uncontrolled eating in adolescents. These findings point to underlying vulnerabilities rather than emotional resilience, warranting further investigation. In contrast, healthy participants also showed automatic avoidance of low‐calorie foods, correlating with the Drive for Thinness and Maturity Fears scales of the EDI‐2, reflecting preoccupations with weight loss, body shape, and adult responsibilities, possibly indicating concerns about inadequate nourishment or a subconscious effort to suppress appetite and control body size (Vartanian and Herman 2006).

Combining all these elements, how can we explain the perceptual prioritisation and implicit avoidance of high‐calorie foods in AN‐R? This phenomenon likely arises from the interplay of distinct neural and cognitive mechanisms involved in threat detection and emotion regulation. Neuroimaging studies show that individuals with AN activate distinct brain regions for processing high‐ and low‐calorie foods, particularly emphasising areas linked to emotional processing (Ellison et al. 2004; Rothemund et al. 2011). This supports the “vigilance‐avoidance” mechanism seen in anxiety disorders (Bar‐Haim et al. 2007), where heightened sensitivity to potential threats is followed by avoidance to regulate distress (Browning et al. 2010; Cisler and Koster 2010). In the case of AN, high‐calorie foods are perceptually prioritised due to their strong association with fears surrounding weight gain and loss of control over eating. This study is the first to provide novel evidence that high‐calorie foods in AN‐R are both perceptually prioritised and avoided at the implicit level, suggesting that this initial hypervigilance is followed by automatic avoidance, likely serving as an emotion regulation strategy to reduce distress (Giel et al. 2011). While this response is adaptive in the short term, it becomes maladaptive over time, reinforcing dietary restraint and disordered eating behaviours (Cowdrey et al. 2013). Ultimately, the heightened perceptual dominance of high‐calorie foods, followed by a subconscious avoidance, may contribute to the persistence and severity of AN‐related eating behaviours and the emotional distress associated with them.

While this study provides valuable insights into perceptual and post‐perceptual implicit biases towards food in AN‐R, some limitations should be considered. The cross‐sectional design prevents causal inferences, highlighting the need for longitudinal studies to track how food‐related biases evolve and respond to treatment. Additionally, external factors like meal context, current hunger or satiety states, emotional state, and social environment may influence automatic food responses, warranting further investigation. Although previous findings suggest that hunger may differentially modulate visual processing, results remain contradictory, depending on the level of awareness (Weng et al., 2019; Ciorli et al. 2024), thus, future research should control for participants' hunger or satiety states as well. While focusing on the restricting subtype provides valuable information, extending research to the binge/purging subtype is essential for a comprehensive understanding of AN's neurocognitive mechanisms. Finally, the exclusive inclusion of female participants limits the generalisability of our findings to males and gender‐diverse individuals with AN.

Future research must integrate neuroimaging and behavioural measures to explore early visual processing and identify key neural circuits involved in food avoidance. Such research is essential for pinpointing intervention targets that could lead to more effective treatments. Longitudinal studies should be conducted to uncover biomarkers that allow for personalised approaches. By targeting interoceptive awareness, emotional regulation, and food‐related anxiety, as well as the subconscious pathways identified in this study, these findings could inform more focused and effective interventions, improving treatment outcomes for AN and offering a path towards more tailored and impactful approaches to managing the disorder.

## Conflicts of Interest

The authors declare no competing interests.

## Supporting information

Supporting Information S1

## Data Availability

The data that support the findings of this study are available from the corresponding author upon reasonable request.
