# Supplementary material for: What the Eye Sees, the Mind Rejects: Implicit Visual Processing of Food Images in Anorexia Nervosa
Source: Eur Eat Disord Rev. 2025 May 19;33(5):1099–110. doi: 10.1002/erv.3210 (PMC12319129; doi:10.1002/erv.3210)
Supplement: Supplementary file 1 — Supporting Information S1 [file ERV-33-1099-s001.docx]

**Section 1**

**Materials and methods: Clinical Assessment**

*1.1 The Eating Disorder Inventory (EDI-2)*

ED-related psychopathology was evaluated with the validated Italian version of the Eating Disorder Inventory-2 (EDI-2; Rizzardi et al., 1995). EDI-2 is a self-report measure employed to assess cognitive aspects and behaviors associated with Eating Disorders (EDs) such as AN, Bulimia Nervosa (BN), and Binge Eating Disorder (BED) (Garner, 1991; Garner et al., 1983). It is reliable, valid, and often used for research purposes. It consists of ninety-one questions distributed on eleven subscales, with items to be rated on a 6-point Likert scale ranging from 1 (never) to 6 (always). The first three scales (drive for thinness, BN, and body dissatisfaction) index the main symptoms of EDs, and the remaining scales measure psychological aspects such as low self-esteem, perfectionism, interpersonal distrust, fear of adulthood, disturbed interoceptive awareness, ascetics, distorted impulse regulation, and social insecurity.

*1.2 The Eating Disorder Examination Questionnaire (EDE-Q)*

The Italian version of EDE-Q (Calugi et al., 2017) was used to assess the occurrence and frequency of the key behavioral features of EDs. EDE-Q is a 28-item self-report questionnaire derived from the Eating Disorder Examination (EDE) interview. It demonstrates strong internal consistency, test-retest reliability, and convergent and discriminant validity (Aardoom et al., 2012; Dahlgren et al., 2017). It includes four subscales: restraint, eating concern, shape concern, and weight concern, as well as an overall global score. It employs a 6-point Likert scale (0–6) with higher scores indicating higher severity or frequency.

*1.3 Multidimensional Assessment of Interoceptive Awareness-2 (MAIA-2)*

MAIA-2 (Mehling et al., 2018) is a recently developed revised version of the MAIA (Mehling et al., 2012), and it is a multi-dimensional instrument designed to measure interoceptive sensibility (Garfinkel et al., 2015). It consists of thirty-seven items designed to assess eight concepts: 1) noticing: awareness of uncomfortable, comfortable, and neutral body sensations; 2) not-distracting: tendency not to ignore or distract oneself from sensations of pain or discomfort; 3) not-worrying: tendency not to worry or experience emotional distress with sensations of pain or discomfort; 4) attention regulation: ability to sustain and control attention to body sensations; 5) emotional awareness: awareness of the connection between body sensations and emotional states; 6) self-regulation: ability to regulate distress by attention to body sensations; 7) body listening: active listening to the body for insight; and 8) trusting: experience of one's body as trustworthy and safe. Participants must rate each item on a 6-point Likert scale, ranging from never (0) to always (5). An Italian version was employed.

*1.4 Body Shape Questionnaire-34 (BSQ-34)*

The BSQ-34 (Cooper et al., 1987) is widely used for assessing cognitively based body dissatisfaction. It demonstrates strong test-retest reliability, good internal consistency, and convergent validity compared to other body dissatisfaction measures in clinical and nonclinical populations. It is a thirty-four-item measure in which participants rate the frequency of negative body-related thoughts on a Likert 6-point Likert scale ranging from 1 (never) to 6 (always) over the last four weeks. This study used the Italian version of the BSQ-34 to assess body image (Marzola et al., 2022).

*1.5 State-Trait Anxiety Inventory (STAI)*

The STAI (Spielberger et al., 1983) is a psychological assessment tool used to measure anxiety, possibly influencing interoception sensitivity. It consists of 40 self-report items rated on a 4-point Likert scale. The inventory evaluates two types of anxiety: state anxiety (STAI-X1), which measures anxiety related to specific events, and trait anxiety (STAI-X2), which assesses the general tendency to experience anxiety. For state anxiety, participants rate their feelings of anxiety "right now" on a scale from 1 (not at all) to 4 (very much so). For trait anxiety, participants indicate the frequency of their anxiety experiences on a scale from 1 (rarely) to 4 (always). This study used the Italian version of the STAI (Pedrabissi & Santinello, 1989).

*1.6 Beck Depression Inventory-II (*BDI-II*)*

The BDI-II (Beck et al., 1996) is a 21-item inventory designed to assess depressive symptoms and their severity, classified as representing ‘none, mild, moderate and severe’ depression. Each item is scored on a scale from 0 to 3. The Italian version of the BDI-II (Montano & Flebus, 2006) was employed here. A revised version of the original Beck Depression Inventory (BDI), the BDI-II, aligns with the DSM-IV criteria for depression and is widely considered one of the most effective tools for distinguishing between non-depressed, moderately depressed, and severely depressed individuals. The BDI-II is known for its strong psychometric properties, including high test-retest reliability and internal consistency (Beck et al., 1996).

**Section 2**

**Results: Clinical Assessment**

*2.1 The Eating Disorder Inventory (EDI-2)*

The AN-R group displayed significantly (*p* < .001) higher scores on nine (out of eleven) scales compared to the HC group: Drive for Thinness (AN-R group mean score = 14.07, SD = 6.34; HC group mean score = 3.14, SD = 5.08), Body Dissatisfaction (AN-R group mean score = 14.75, SD = 5.64; HC group mean score = 6.33, SD = 5.32), Ineffectiveness (AN-R group mean score = 14.18, SD = 5.56; HC group mean score = 4.03, SD = 4.37), Interpersonal Distrust (AN-R group mean score = 7.82, SD = 4.36; HC group mean score = 3.06, SD = 3.82), Interoceptive Awareness (AN-R group mean score = 12.79, SD = 6.89; HC group mean score = 4.36, SD = 5.92), Maturity Fears (AN-R group mean score = 9.25, SD = 5.54; HC group mean score = 4.44, SD = 4.05), Asceticism (AN-R group mean score = 7.79, SD = 4.78; HC group mean score = 2.50, SD = 1.80), Impulse Regulation (AN-R group mean score = 6.14, SD = 4.84; HC group mean score = 2.30, SD = 4.08), and Social Insecurity (AN-R group mean score = 10.39, SD = 3.38; HC group mean score = 4.06, SD = 3.77).

*2.2 The Eating Disorder Examination Questionnaire (EDE-Q)*

The AN-R group displayed significantly (*p* < .001) higher scores on all the scales compared to the HC group: Restraint (AN-R group mean score = 3.19, SD = 1.70; HC group mean score = 1.09, SD = 1.07), Eating Concern (AN-R group mean score = 3.14, SD = 1.25; HC group mean score = 0.89, SD = 1.10), Shape Concern (AN-R group mean score = 4.54, SD = 1.05; HC group mean score = 1.84, SD = 1.58), Weight Concern (AN-R group mean score = 3.92, SD = 1.28; HC group mean score = 1.39, SD = 1.35), Global Score (AN-R group mean score = 3.70, SD = 1.09; HC group mean score = 1.30, SD = 1.12).

*2.3 Multidimensional Assessment of Interoceptive Awareness-2 (MAIA-2)*

The AN-R group had significantly (*p* < .05) lower scores on five (out of eight) scales compared to the HC group: Not Worrying (AN-R group mean score = 2.00, SD = 0.86; HC group mean score = 2.56, SD = 0.98), Attention Regulation (AN-R group mean score = 1.60, SD = 0.71; HC group mean score = 2.43, SD = 0.94), Self-Regulation (AN-R group mean score = 1.60, SD = 1.01; HC group mean score = 2.32, SD = 1.16), Body Listening (AN-R group mean score = 1.50, SD = 0.93; HC group mean score = 2.34, SD = 1.38), and Trusting (AN-R group mean score = 1.00, SD = 0.92; HC group mean score = 3.02, SD = 1.34).

*2.4 Body Shape Questionnaire-34 (BSQ-34)*

The AN-R group displayed significantly (*p* < .001) higher scores on the Total Score compared to the HC group: (AN-R group mean score = 118.27, SD = 48.62; HC group mean score = 78.94, SD = 33.08).

*2.5 State-Trait Anxiety Inventory (STAI)*

The AN-R group displayed significantly (*p* < .001) higher scores on the two scales, compared to the HC group, namely State Anxiety (AN-R group mean score = 57.89, SD = 11.91; HC group mean score = 41.19, SD = 11.15) and Trait Anxiety (AN-R group mean score = 59.86, SD = 9.73; HC group mean score = 47.22, SD = 11.00).

2.6 *Beck Depression Inventory-II (*BDI-II*)*

The AN-R group showed significantly (*p* < .001) higher scores on the three scales compared to the HC group, that is, Cognitive (AN-R group mean score = 16.61, SD = 6.53; HC group mean score = 6.19, SD = 5.18), Somatic (AN-R group mean score = 13.65, SD = 5.47; HC group mean score = 5.64, SD = 4.49), and Total Score (AN-R group mean score = 30.26, SD = 11.59; HC group mean score = 11.83, SD = 9.40).

**References**

Aardoom, J. J., Dingemans, A. E., Slof Op't Landt, M. C., & Van Furth, E. F. (2012). Norms and discriminative validity of the Eating Disorder Examination Questionnaire (EDE-Q). *Eating Behaviors*, *13*(4), 305-309. <https://doi.org/10.1016/j.eatbeh.2012.09.002>

Beck, A. T., Steer, R. A., Ball, R., & Ranieri, W. (1996). Comparison of Beck Depression Inventories -IA and -II in psychiatric outpatients. *Journal of Personality Assessment*, *67*(3), 588-597. <https://doi.org/10.1207/s15327752jpa6703_13>

Calugi, S., Milanese, C., Sartirana, M., El Ghoch, M., Sartori, F., Geccherle, E., Coppini, A., Franchini, C., & Dalle Grave, R. (2017). The Eating Disorder Examination Questionnaire: reliability and validity of the Italian version. *Eat Weight Disord*, *22*(3), 509-514. <https://doi.org/10.1007/s40519-016-0276-6>

Cooper, P. J., Taylor, M. J., Cooper, Z., & Fairbum, C. G. (1987). The development and validation of the body shape questionnaire. *International Journal of Eating Disorders*, *6*(4), 485-494.

Dahlgren, C. L., Stedal, K., & Ro, O. (2017). Eating Disorder Examination Questionnaire (EDE-Q) and Clinical Impairment Assessment (CIA): clinical norms and functional impairment in male and female adults with eating disorders. *Nord J Psychiatry*, *71*(4), 256-261. <https://doi.org/10.1080/08039488.2016.1271452>

Garfinkel, S. N., Seth, A. K., Barrett, A. B., Suzuki, K., & Critchley, H. D. (2015). Knowing your own heart: distinguishing interoceptive accuracy from interoceptive awareness. *Biological Psychology*, *104*, 65-74. <https://doi.org/10.1016/j.biopsycho.2014.11.004>

Garner, D. M. (1991). *Eating Disorders Inventory-2: Professional Manual*. Psychological Assessment Resources.

Garner, D. M., Olmstead, M. P., & Polivy, J. (1983). Development and validation of a multidimensional Eating Disorder Inventory for Anorexia Nervosa and Bulimia. *International Journal of Eating Disorders*, *2*(2), 15-34.

Marzola, E., Martini, M., Longo, P., Toppino, F., Bevione, F., Delsedime, N., Abbate-Daga, G., & Preti, A. (2022). Psychometric properties of the Italian body shape questionnaire: an investigation of its reliability, factorial, concurrent, and criterion validity. *Eat Weight Disord*, *27*(8), 3637-3648. <https://doi.org/10.1007/s40519-022-01503-6>

Mehling, W. E., Acree, M., Stewart, A., Silas, J., & Jones, A. (2018). The Multidimensional Assessment of Interoceptive Awareness, Version 2 (MAIA-2). *PloS One*, *13*(12), e0208034. <https://doi.org/10.1371/journal.pone.0208034>

Mehling, W. E., Price, C., Daubenmier, J. J., Acree, M., Bartmess, E., & Stewart, A. (2012). The Multidimensional Assessment of Interoceptive Awareness (MAIA). *PloS One*, *7*(11), e48230. <https://doi.org/10.1371/journal.pone.0048230>

Montano, A., & Flebus, G. B. (2006). Presentation of the beck depression inventory – second edition (BDI-II-II): confirmation of bifactorial structure in a sample of the Italian population. *Psicoterapia Cognit Comportamentale*, *12*, 67-82.

Pedrabissi, L., & Santinello, M. (1989). *Verifica della validità dello STAI forma y di Spielberger* (Vol. 11). Giunti, Organizzazioni Speciali.

Rizzardi, M., Trombini Corazza, E., & G., T. (1995). *Manuale: EDI-2 eating disorder inventory--2*. Organizzazioni Speciali.

Spielberger, C. D., Gorsuch, R. L., Lushene, R., Vagg, P. R., & Jacobs, G. A. (1983). *Manual for the State-Trait Anxiety Inventory*. Consulting Psychologists Press.
